# Evaluation of China’s Environmental Pressures Based on Satellite NO_2_ Observation and the Extended STIRPAT Model

**DOI:** 10.3390/ijerph16091487

**Published:** 2019-04-26

**Authors:** Yuanzheng Cui, Lei Jiang, Weishi Zhang, Haijun Bao, Bin Geng, Qingqing He, Long Zhang, David G. Streets

**Affiliations:** 1 Institute of Land and Urban-rural Development, Zhejiang University of Finance and Economics, Hangzhou 310018, China; cuiyuanzheng@163.com (Y.C.); gengbin_zjcjdx@126.com (B.G.); 2 School of Economics, Zhejiang University of Finance and Economics, Hangzhou 310018, China; 3 School of Geographic and Environmental Sciences, Tianjin Normal University, Tianjin 300387, China; 4 State Key Joint Laboratory of Environment Simulation and Pollution Control (SKLESPC), School of Environment, Tsinghua University, Beijing 100084, China; 5 School of Public Administration, Zhejiang University of Finance and Economics, Hangzhou 310018, China; baohaijun@zufe.edu.cn; 6 School of Resources and Environmental Engineering, Wuhan University of Technology, Wuhan 430070, China; qqhe@link.cuhk.edu.hk; 7 Business School, Xinyang Normal University, Xinyang 464000, China; hbzhanglong876@163.com; 8 Energy Systems Division, Argonne National Laboratory, Argonne, IL 60439, USA; dstreets@anl.gov

**Keywords:** nitrogen dioxide pollution, extended STIRPAT model, urban environmental pressures, Chinese cities, satellite observations

## Abstract

China’s rapid urbanization and industrialization have affected the spatiotemporal patterns of nitrogen dioxide (NO_2_) pollution, which has led to greater environmental pressures. In order to mitigate the environmental pressures caused by NO_2_ pollution, it is of vital importance to investigate the influencing factors. We first obtained data for NO_2_ pollution at the city level using satellite observation techniques and analyzed its spatial distribution. Next, we introduced a theoretical framework, an extended stochastic impacts by regression on population, affluence, and technology (STIRPAT) model, to quantify the relationship between NO_2_ pollution and its contributing natural and socio-economic factors. The results are as follows. Cities with high NO_2_ pollution are mainly concentrated in the North China Plain. On the contrary, southwestern cities are characterized by low NO_2_ pollution. In addition, we find that population, per capita gross domestic product, the share of the secondary industry, ambient air pressures, total nighttime light data, and urban road area have a positive impact on NO_2_ pollution. In contrast, increases in the normalized difference vegetation index (NDVI), relative humidity, temperature, and wind speed may reduce NO_2_ pollution. These empirical results should help the government to effectively and efficiently implement further emission reductions and energy saving policies in Chinese cities in a bid to mitigate the environmental pressures.

## 1. Introduction

The spatial patterns of nitrogen dioxide (NO_2_) pollution in China have changed dramatically due to the rapid development of urbanization and industrialization in recent decades [1]. The overuse of fossil fuels by industrial production, transportation, thermal power plants, and residential use have led to a tremendous increase in NO_2_ pollution in the air. China has already become one of the most severely NO_2_ polluted countries in the world [1,2]. Moreover, the average annual surface NO_2_ concentration in many cities now far exceeds the “air quality standards” (40 μg/m^3^) issued by the World Health Organization in 2005 [3,4]. Exposure to high concentrations of NO_2_ can directly affect human health through decreased lung function and increased respiratory disease [5].

One of the major contributors to China’s environmental degradation are nitrogen oxides (NOx = NO_2_ + NO), which play a vital role in tropospheric atmospheric chemistry and climate change, including producing ozone, aerosols, and acid rain, as well as changing the radiative forcing [6]. The sources of NO_x_ are derived from both anthropogenic emissions (mainly thermal power plants, transportation, industries, and residential use) and natural emissions (including lightning, open fires, and soil) [7]. NASA’s INTEX-B emissions inventory in 2006 showed that China’s NO_x_ emissions accounted for 57% of the total in Asia [8]. Thus, NO_x_ has been listed in the emission reduction target of air pollutants since the 12th five-year plan proposed by the Chinese central government [9]. In addition, the average annual NO_2_ concentration has become one of the important indicators of the performance evaluation of local governments across the country in recent years [10].

Satellite observation technology has the advantages of macroscopic dynamic monitoring in real-time and large-scale coverage, and its observation data can reflect ground-level human activities and environmental change. Tropospheric NO_2_ vertical column densities (VCDs) retrieved from a series of satellite instruments have been widely applied to study NO_2_ pollution over China [1,2,11,12,13]. Previous studies also showed that both changes in the tropospheric NO_2_ columns and changes in surface NO_2_ concentrations were closely correlated, indicating the possibility of using the satellite-observed tropospheric NO_2_ to detect the changes in NO_2_ pollution on the ground over China [14,15]. NO_2_ is one of the main air quality indicators that reflect the environmental quality of each city. Local governments need to pay more attention to the reduction of NO_2_ pollution. Thus, it is of great significance to quantify the driving factors of NO_2_ pollution at the city level while monitoring the NO_2_ pollution in different ways.

By reviewing the existing literature, we can see that a growing number of studies has explored the driving factors of air quality in China. For example, Zhao, et al. [16] found that urbanization had a negative relationship with air quality in Chinese cities. Lyu, et al. [17] applied an index decomposition method to analyze the driving forces of air pollution emissions from 1997 to 2012, indicating that economic growth and energy intensity are the most important key factors affecting air quality in China. Zhang, et al. [18] analyzed the driving factors of ambient air quality in Beijing. Unlike classical regression models, some researchers took into account spatial effects in the models. Specifically, spatial econometric regression models have been applied to detect the relationship between air quality and socio-economic factors in China [19,20,21,22]. The previous empirical studies focused on the driving factors of particulate matter, while other studies found that urban development, civilian vehicles, power usage, population density, built-up areas, and coal consumption have close relationships with the NO_2_ pollution levels [6,15,Huang, et al. [23]]. However, these studies merely focused on the socio-economic driving factors previously mentioned above, with less attention paid to the natural factors, including the meteorological conditions and vegetation index that also affect the NO_2_ concentrations [5,24]. Both natural factors and socio-economic factors exert a large influence on environmental health.

One of the major sources of NO_2_ pollution is fuel burning [1]. However, the availability of energy consumption data at the city level is extremely limited in China. Thus, some researchers found that satellite-observed nighttime light data is a good proxy for describing energy consumption at different regional scales [25,26,27]. Additionally, the normalized difference vegetation index (NDVI) products observed by satellites, which describe the vegetation coverage at high spatial and temporal resolution, are also taken into consideration [20,28]. To sum up, the contribution of this research is threefold. First, we applied a widely-used theoretical framework, namely the STIRPAT model (a stochastic model to study the stochastic impacts by regression on population, affluence, and technology) to examine the driving factors of NO_2_ pollution at the city level. Second, we extended the STIRPAT model by taking into account meteorological factors, namely, ambient air pressure, relative humidity, temperature, and wind speed, in addition to socio-economic factors. Third, we introduced the remote-sensing technique to obtain a series of proxy indicators for economic and natural factors, such as nighttime light data for energy use and NDVI for vegetation coverage to improve the reliability and quality of data, since these data are not available in statistical yearbooks. To conclude, the broad purpose of this study was to apply a theoretical model and introduce remote sensing techniques to comprehensively reveal the driving factors of NO_2_ pollution in Chinese cities. The findings of the research will help us to better understand the forces that drive increasing NO_2_ emissions, and to inform us of how to effectively and efficiently mitigate NO_2_ pollution and improve environmental health.

In this research, we analyzed the spatial distribution of satellite-observed NO_2_ pollution over China by employing spatial statistical methods. By incorporating multi-source remote-sensing satellite observation data, the relationship between NO_2_ pollution and its driving factors, including both natural factors and socio-economic factors, were then quantified by an extended STIRPAT model. Finally, the policy implications are presented. Our results can also help the government make better emission reduction and energy saving policies as well as reduce environmental pressures.

## 2. Methods and Data Sources

### 2.1 Methodology

#### 2.1.1. Spatial Autocorrelation Method

According to Tobler’s first law of geography, “everything is related to everything else, but near things are more related than distant things” [29]. Unlike ordinary correlation, the spatial autocorrelation method is applied to investigate the relationship between a spatial unit and its neighbors. Hence, it is useful to detect the cluster patterns as well as identify the spatial hot-spots over regions [30]. In this work, the global Moran’s I measure was employed to examine the spatial patterns of the annual mean tropospheric NO_2_ for all the prefectural-level cities (including four municipalities, Beijing, Tianjin, Shanghai, and Chongqing) in mainland China. The global Moran’s I measures the degree of clustering or dispersion for the whole study area. However, the results cannot identify the exact regions showing the clustering patterns. Therefore, we subsequently perform hot spot analysis (Getis-Ord Gi* statistic) to detect the local regions with the hot spots and cold spots of annual mean tropospheric NO_2_.

Global Moran’s I is expressed as follows.

(1) $$I=\;\frac{\sum_{i=1}^{n}\sum_{j=1}^{n}w_{ij}\left(x_{i}-\bar{x}\right)\left(x_{j}-\bar{x}\right)}{S^{2}\sum_{i=1}^{n}\sum_{j=1}^{n}w_{ij}}$$

where *x_i_* is the attribute value for a spatial unit, namely prefectural-level city *i. w_i,j_* is the spatial weight between city *i* and city *j* by calculating the inverse Euclidean distance (the longer distance indicates the smaller spatial weight value). Specifically, *w_i,j_* equals 1/d*_i,j_* where d*_i,j_* denotes the distance of each pair of city *i* and city *j*. *n* is equal to the total number of cities and:

(2) $$S^{2}=\frac{1}{n}\overset{n}{\underset{i=1}{\sum }}{\left(x_{i}-\bar{x}\right)}^{2}$$

(3) $$\bar{x}=\frac{1}{n}\overset{n}{\underset{i=1}{\sum }}x_{i}$$

Global Moran’s I is positive, indicating that NO_2_ pollution at the city level tends to be similar and show clustered spatial patterns. Moran’s I is negative indicating that NO_2_ pollution at the city level presents dispersed spatial patterns. NO_2_ pollution is randomly distributed when Moran’s I is zero. The results are valid only when the statistical significance of the p-value is smaller than 0.05 at the 95% confidence level.

The hot-spot analysis method is able to calculate the Getis-Ord Gi* statistic of tropospheric NO_2_ data at the city level. It is given below.

(4) $$G_{j}^{*}=\frac{\sum_{i=1}^{n}w_{i,j}x_{i}\;-\bar{x}\sum_{i=1}^{n}w_{i,j}\;}{S\sqrt{\frac{\left[n\sum_{i=1}^{n}w_{i,j}^{2}-{\left(\sum_{i=1}^{n}w_{i,j}\;\right)}^{2}\right]}{n-1}}}$$

The statistically significant positive Gi* statistic indicates the clustering of high values (hot spot) and the statistically significant negative Gi* statistic indicates the clustering of low values (cold spot).

#### 2.1.2. Econometric Methods

In order to elucidate environmental degradation and to specify the factors influencing environmental impacts, [31] were the first to propose the IPAT model (*I* = *PAT*). The idea of the IPAT model is that environmental impacts (I) are a function of three main driving factors, namely, population size (P), affluence (A), and technology level (T). The environmental impacts have been previously addressed for certain pollutants, for example, CO_2_ emissions [32,33,34], SO_2_ emissions [35,36], energy consumption [37,38], wastewater discharges [39,40], etc. Moreover, PAT denotes three main socio-economic driving factors affecting environmental quality. The IPAT model is an easy way to investigate the factors of environmental pressures caused by anthropogenic activities.

We define the IPAT model as follows:

(5) I=P·A·T

where *I* denotes environmental impacts, which are measured by the tropospheric NO_2_ columns in this research. Generally, the higher the NO_2_ value, the worse the environmental degradation, and the greater the environmental pressure. *P* represents the size of the population of a region, while *A* denotes the affluence level of the region, usually measured by per capita income. The increases in population and affluence levels lead to the generation of many pollutants, with the subsequent degradation of the environment. *T* denotes the technology level, which is able to reverse the negative effects of population and affluence on the environment.

Empirically, the IPAT model is usually reformulated into a stochastic model, namely, the STIRPAT model (a stochastic model, named for the stochastic impacts by regression on population, affluence, and technology) [41]. It is expressed as follows:

(6) $$I=aP^{b}A^{c}T^{d}e$$

where *a* is a constant and *b*, *c*, and *d* denote the coefficients of the variables of population, affluence, and technology levels, respectively. *e* is the error term.

In order to implement the IPAT model, we empirically transform Equation (1) into a linear model, by means of taking logarithms. One advantage is that logarithmic transformation is able to reduce the possible issue of heterogeneity. Then, it can be written as follows:

(7) LnI=Lna+bLnP+cLnA+dLnT+Lne

where *Ln* denotes natural logarithms. All other variables are the same as in Equation (2).

In this research, we employed a panel data set of 243 Chinese cities from 2005 to 2012 to discover the factors influencing NO_2_ pollution. In the panel data setting, the model can be rewritten as follows.

(8) $$LnI_{it}=\alpha +\beta_{1}LnP_{it}+\beta_{2}LnA_{it}+\beta_{3}LnT_{it}+\varepsilon_{it}$$

where subscript *i* and t denote the *i_th_* city of the year *t*. In addition, for simplicity we replaced the constant term *Lna* with α, and *Lne* with ε. *β* is used to denote the coefficients to be estimated.

Environmental pressures are not only driven by population, affluence, and technology but also other socio-economic influencing factors, even natural and meteorological factors. In order to fully understand the driving factors of NO_2_ pollution at the city level, we also took into account a set of explanatory variables. Hence, the extended STIRPAT model can be written as follows.

(9) $$LnI_{it}=\alpha +\beta_{1}LnP_{it}+\beta_{2}LnA_{it}+\beta_{3}LnT_{it}+\sum_{j=1}^{k}\beta_{j}x_{it}+\mu_{i}+\varepsilon_{it}$$

where *x* denotes a set of explanatory variables, namely, nighttime light (*NTL*), normalized difference vegetation index (*NDVI*), urban road area (*Road*), and natural and meteorological factors, namely, atmospheric pressure (Pres), relative humidity (*Humi*), temperature (*Temp*), and wind speed (*WS*), which will be discussed later. *β_j_* denotes the estimated coefficients to these explanatory variables. Besides, *μ_i_* denotes city-specific and time-invariant variables that are not included in the STIRPAT model. It can be treated as a fixed effects or random effects model. However, for the case of the Chinese cities, a city-specific fixed effects model was likely to be better fitted than the random effects model. The reason may lie in two aspects. One is that the unobserved disturbance in the model may be correlated with the explanatory variables. The other is that heterogeneity between cities cannot be random. Therefore, the omission of the fixed effects may lead to biased results. In order to verify the hypothesis in support of the fixed effects model, we performed a Hausman test to examine if the fixed effects model was better than the random effects model.

#### 2.1.3. Variables

The dependent variable in the regression models was the tropospheric NO_2_ VCDs at the city level, which is employed to measure the level of pollution in a region. In order to better understand what causes NO_2_ pollution, we introduced an extended STIRPAT model, including population, affluence, technology level, and a set of other explanatory variables mentioned above, which will be discussed one-by-one.

Population (*PopDen*). An increase in population leads to demand for a larger amount of various resources, for example, energy, and thus generates an increased amount of pollutants. As a consequence, the ever-growing population is a big challenge for the environmental carrying capacity. Specifically, China, the most populous country with approximately 1.4 billion people, needs a wide variety of resources to support the demand for a better standard of living as China’s economy continues to rapidly increase. However, China has also witnessed a series of severe environmental problems, notably air pollution. Hence, it is hypothesized that population has a positive impact on NO_2_ pollution.

Affluence (*PCGDP*). Affluence is a major influencing factor that generally worsens environmental quality. The main reason is that as income levels gradually increase, a large number of high-energy-consuming industrial products and services are consumed, for example, vehicles, air-conditioners, and central heating systems. As a result, various air pollutant emissions result. Hence, it is assumed that affluence is positively correlated with NO_2_ pollution. In this research, the per capita GDP of a city was used to measure local affluence.

Technology (*STRatio*). Technological progress is the most effective way to reduce emissions and improve environmental quality. In this research, we employed the ratio of the secondary industry to the tertiary industry to denote the level of technologies. This is because the industrial sector is the largest pollutant emitter. In the rapid process of industrialization, China has witnessed the large-scale expansion of all industrial sectors in a bid to realize the goal of economic growth, but at the expense of the environment. In the early stage of economic development, resource-extensive industrial sectors with low technology and added value dominated the economy. As technological progress continued, China is facing a transition from the secondary industry to the tertiary industry, which is characterized by a higher level of technology and higher added value. This industrial upgrade is consistent with the technological changes that accompany economic development. Hence, the greater the ratio of secondary industry to tertiary industry, the lower the technology level, and vice versa. We hypothesized that this has a positive effect on NO_2_ pollution.

When analyzing the factors influencing the environmental impact, we also take into account a set of other socio-economic factors as well as natural factors in the regression model, including nighttime light (*NTL*), normalized difference vegetation index (*NDVI*), urban road area (*Road*), ambient pressure (Pres), relative humidity (Humi), temperature (Temp) and wind speed (WS), in addition to population, affluence, and technology contained in the extended STIRPAT model.

Nighttime light (NTL). Rapid urbanization and industrialization in China have consumed a lot of energy resources. Profligate burning of fossil fuels releases large quantities of NOx into the atmosphere, thus deteriorating the local environment. In recent decades, China has consumed almost half of the coal in the world, and about 70% of China’s industrial and residential energy consumption is supplied by coal burning [42]. In recent literature [43,44,45,46], satellite-observed nighttime light data have been widely used to explore the levels of socio-economic development. Additionally, the total annual nighttime light data have been closely correlated with energy consumption [25,26]. Generally, the greater the energy consumption, the more NOx is emitted, and the more NO2 in the atmosphere. To sum up, in this study we used the total nighttime light value as a proxy to describe energy consumption at high resolution and hypothesize that it has a positive effect on NO2 pollution.

Urban road area (*Road*). Vehicles in cities are one of the major sources of NO_2_ pollution [8]. According to the Multi-resolution Emission Inventory for China (MEIC) inventory, NO_x_ emitted from vehicles accounted for about 25.4% of total anthropogenic emissions in 2010 over China [47]. In recent years, China has witnessed a rapid increase in the number of vehicles as a consequence of economic growth and the development of the transportation industry, heavily dependent on the building of the urban road. In other words, the expansion of the urban road area has a greater capacity to allow more vehicles and contribute to the development of the transportation industry, which results in increased pollution. Hence, we hypothesize that the expansion of the urban road area is positively associated with NO_2_ pollution.

The normalized difference vegetation index (NDVI) can accurately reflect the surface vegetation coverage. Cities with higher NDVI values are experiencing lighter environmental pressures and the vegetation is well-known to reduce the air pollutants in the air. Therefore, a higher NDVI index may exert a negative effect on NO_2_ pollution.

### 2.2. Data Sources

The ozone monitoring instrument (OMI) onboard the EOS-Aura satellite was launched on 15 July 2004. The satellite flies in a sun-synchronous orbit with the local equator crossing time around 13:40. The OMI pixel size varies from 13 × 24 km^2^ at nadir to 40 × 250 km^2^ at the outermost fields of view. The detailed tropospheric NO_2_ columns retrieval algorithms were reported by Boersma, et al. [48]. In this research, the daily level-2 DOMINO v2 swath product dataset (http://www.temis.nl/airpollution/NO2.html) from the Royal Netherlands Meteorological Institute (KNMI) was applied. We mapped the daily swath data into to a 0.125° × 0.125° grid dataset using the area-weighted averages interpolation method. We only included valid pixels with a cloud radiance fraction <50% and removed pixels that were affected by the row anomaly. The daily NO_2_ VCDs were averaged into monthly NO2 VCD values and then averaged into annual NO_2_ VCD values in this study. The detailed description of the satellite data processing is the same as in Cui, Zhang, Bao, Wang, Cai, Yu and Streets [45]. The uncertainties of tropospheric NO2 column data are ±30% (relative error) and 0.7 × 1015 molecules cm^−2^ (absolute error) [48].

Other ancillary data sources related to the study of contributing factors are as follows: (1) the nighttime light data from 2005 to 2012 were retrieved from the the Defense Meteorological Satellite Program’s Operational Linescan System (DMSP/OLS) satellite instrument. The episodic events (fires, gas flares, volcanoes or aurora) of NTL were removed and further refined to be consistent with the time series developed by Zhang, et al. [49]. The total annual nighttime light data at the city level was then calculated; (2) normalized difference vegetation index (NDVI) data were provided by the MODIS Version 5 NDVI Level 3 monthly product with a spatial resolution of 1 km (https://lpdaac.usgs.gov/dataset_discovery/modis/modis_products_table/myd13a3). To ensure a better description of vegetation conditions at the city level, we only selected the NDVI value during the growing season (March–October) to process into the annual mean NDVI dataset [50]; (3) the ground-based meteorological variables, including relative humidity, temperature, wind speed, and pressure were obtained from the National Meteorological Information Center of China Meteorological Administration (http://data.cma.cn/). We interpolated the daily meteorological station data to 3 km spatial resolution using an inverse-distance-weighted (IDW) method, and then averaged them into an annual mean dataset; (4) a set of yearly socio-economic indices for cities from 2005 to 2012, including gross domestic product per capita, population, primary, secondary and tertiary industrial outputs, built-up areas and road areas from the China Statistical Yearbook [51] and the China City Statistical Yearbook [52]. In this work, the tropospheric NO_2_, NDVI, and NTL data and meteorological parameters at the prefectural level were retrieved according to China’s administrative boundaries. Due to the consistency of the DMSP-OLS nighttime light data, we chose the study period of 2005–2012 for this research.

The meteorological parameters, including ambient air pressure (Pres), temperature (Temp), humidity (Humi), and wind speed (WS), are also important factors that affect the tropospheric NO_2_ in different regions. Increasing the temperature can enhance photochemical reactions and reduce the lifetime of NO_2_ in the atmosphere; higher relative humidity causes lower tropospheric NO_2_ by promoting the conversion rate from NO_x_ to secondary aerosols; wind speed affects the rate of diffusion and dilution of pollutants in the atmosphere; and higher pressure increases NO_2_ levels by enhancing the stability of the atmosphere [5]. Therefore, the descriptive statistics of the variables involved in the regression models (mean, standard deviation (Std. Dev), minimum (Min) and maximum (Max)) are summarized in Table 1.

## 3. Results

### 3.1. Spatial Characteristics of NO_2_ Pollution over China

**Figure 1:**
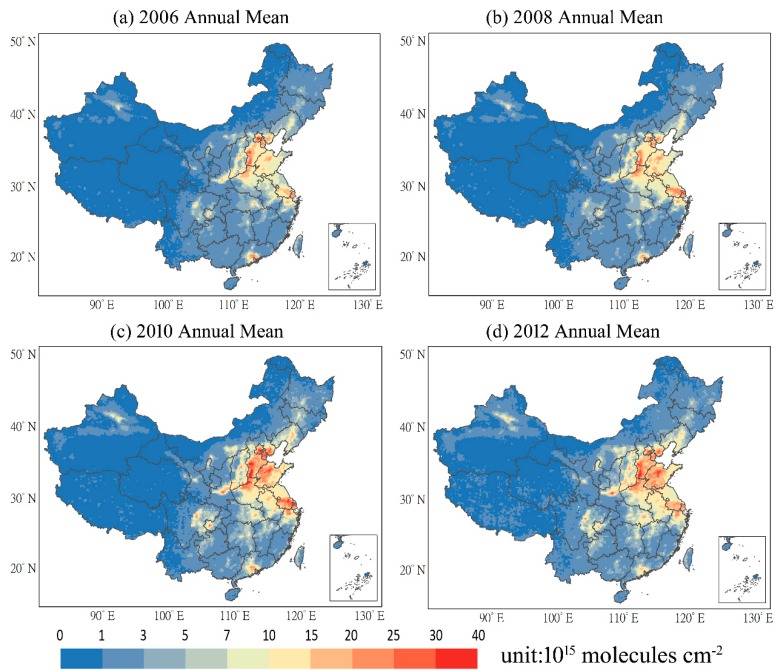
Spatial distribution of annual mean tropospheric NO_2_ over China in 2006, 2008, 2010 and 2012.

Figure 1 shows the spatial distribution of the annual mean tropospheric NO_2_ over China in 2006, 2008, 2010, and 2012. The severe NO_2_ pollution regions not only covered the traditional urban clustering regions, namely, the North China Plain (NCP, including Beijing, Tianjin, Hebei, Henan and Shandong provinces), the Yangtze River Delta (YRD), and the Pearl River Delta (PRD), but also include Shanxi Province, the Chengyu city-cluster and the Guanzhong city-cluster. As shown in Figure 1, the polluted regions (grids in color from light yellow to red, exceeding 7 × 10^15^ molecules cm^−2^) gradually enlarged during this period. Moreover, the NO_2_ pollution of most regions presented an increasing trend in these years, particularly the NCP region, Chengyu city-cluster, and Guanzhong city-cluster. On the other hand, the PRD region showed a decreasing trend of NO_2_ pollution.

**Figure 2:**
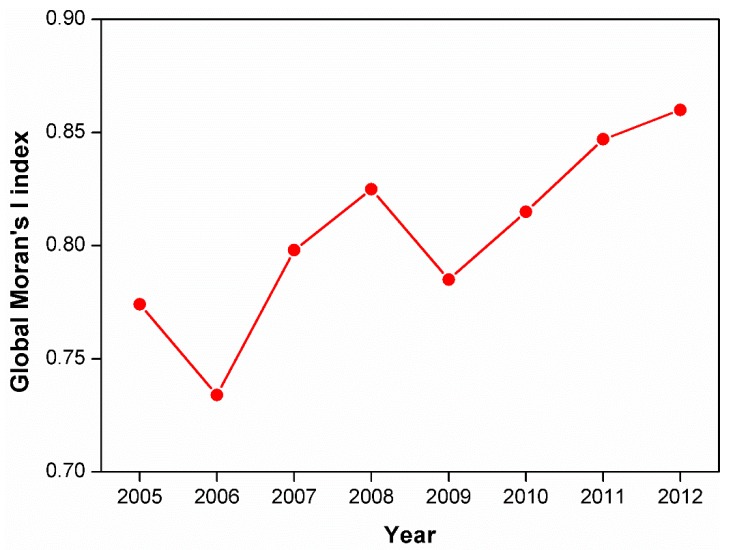
Global Moran’s I values at the city level from 2005 to 2012.

**Figure 3:**
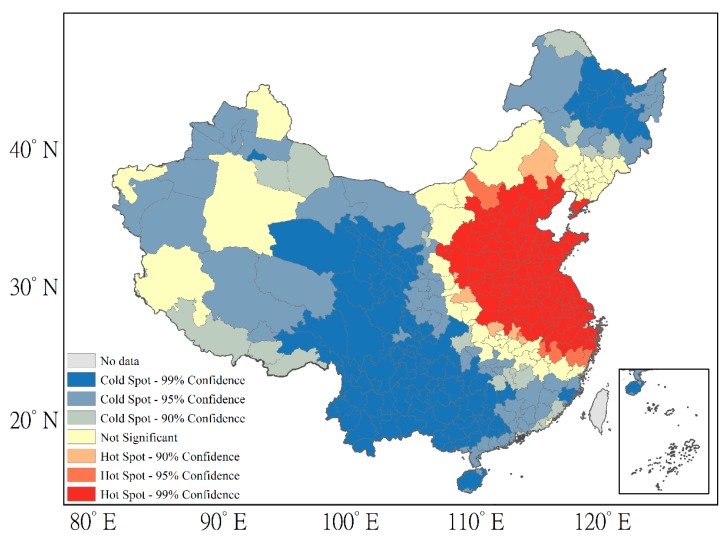
Hot-spot analysis over China in 2012.

Spatial autocorrelation methods were then employed, including the global Moran’s I method and the hot-spot analysis technique, to further quantify the spatial patterns of tropospheric NO_2_ at the city level over China. Euclidean distance and inverse distance conceptualization were chosen for this analysis. Figure 2 shows the results of the application of the global Moran’s I method from 2005 to 2012. Overall, the index exhibited an increasing trend, with two interruptions in 2006 and 2009. The high Moran’s I index values (0.73–0.86) suggest that tropospheric NO_2_ exhibited significant clustering over China during this period. We next employed the hot-spot analysis. As shown in Figure 3, the results of the hot-spot analysis at the city level in 2012 revealed that hot-spots are mainly concentrated in the NCP region and the YRD region, while cold-spots are found in the southwest, mainly in Yunnan, Sichuan, Guizhou and Guangxi provinces.

### 3.2. Regression Model Results

In this subsection, we present and discuss the estimated results of the extended STIRPAT model. However, before addressing these estimations, the Pearson correlation coefficients between variables are reported. These are summarized in Table 2.

We will first present the results of the pooled least squares (PLS) model, since it generally serves as the benchmark model. These results are summarized in Table 3. As shown in Table 3, all explanatory variables were statistically significant at the 1% significance level. The R^2^ statistic indicates that 75.1% of the total variation of the dependent variable, namely, the NO_2_ pollution level, can be explained by these 10 explanatory variables. The results of an F statistic test showed that the null hypothesis of the joint insignificance of all variables can be strongly rejected. In addition, we performed a test for the potential issue of multicollinearity. It was found that the variance inflation factor of each explanatory variable was not greater than 10, indicating that the model did not suffer from the problem. To conclude, the PLS model was well fitted.

Regarding the estimated coefficients, we found that population, per capita GDP, the ratio of the secondary industry to the tertiary industry, total nighttime light data, normalized difference vegetation index, and wind speed were positively correlated with NO_2_ pollution, while urban road area, relative humidity, and temperature had negative impacts on NO_2_ pollution. We did not expect that the relative humidity would have a negative impact. Moreover, its elasticity coefficient was −2.1780 and may be overestimated. Similarly, the wind speed variable was found to be positive, also contradicting our expectations. The major explanation for these unexpected results was that the PLS model ignored city-specific fixed effects, which may have led to biased results. Hence, we conducted an F test and found that the null hypothesis of the joint insignificance of *μ_i_* could be strongly rejected at the 1% significance level. Hence, we concluded that the fixed effects model may be better fitted than the PLS model. The next step was to compare the results of the fixed effects model and the random effects model. The results are summarized in Table 4.

We found that all variables in the fixed effects model were statistically significant at least at the 5% significance level. On the contrary, for the random effects model, the variables of NDVI and urban road area were highly insignificant. In order to determine if the fixed effects model was better fitted than the random effects model, we performed a Hausman test. The test results were in favor of the fixed effects model.

The population variable was found to be positive, in line with our expectations, indicating that an increase in population results in NO_2_ pollution. The coefficient is 0.3874, indicating that an increase in population of 1% within the dataset was associated with a rise in NO_2_ pollution of 0.3874%. Population is mainly attributed to worsening environmental quality, since inhabitants consume a large amount of energy and other resources, and thus generate various pollutants. Although the growth rate of the population of China has been gradually declining during the last decade, more than 1.4 billion people will pose a huge threat to the environment in the long run.

Per capita GDP also had a positive correlation with NO_2_ pollution. We found a positive coefficient of 0.3783, meaning that increases in income levels of 1% within the dataset were associated with increases in NO_2_ pollution of 0.3783%. We also implemented a robustness check, by testing the quadratic term of the per capita income to verify if this generates an inverted U-shaped curve. Specifically, we assumed that the NO_2_ pollution passes a turning point and is then improved as income levels continue to rise. However, we found that the quadratic term was highly insignificant and drew the conclusion that there was an increasingly linear relationship between income and NO_2_ pollution.

Regarding the ratio of secondary industry to tertiary industry, it was found that the impact of this ratio on NO_2_ pollution was significant and positive, as expected. The positive coefficient, 0.1055, indicated that within the dataset an increase in the share of secondary industry to tertiary industry by 1% was associated with increases in NO_2_ pollution of 0.1055%. Secondary industry has long dominated China’s economy, because large-scale industrialization was considered to be the best way to stimulate economic growth and realize common prosperity in the early stage of economic development. As a consequence, secondary industry, the largest pollutant emitter, contributes greatly to the NO_2_ pollution in China. Fortunately, China has been experiencing an industrial upgrade in recent years, and the transition from pollution-intensive secondary industry to tertiary industry is characterized by advanced technology and high added value. Hence, it is predicted that industrial upgrades and technological changes will reduce the negative effects on environmental quality.

Nighttime light data, as a proxy for energy consumption, were also found to have a positive relationship with NO_2_ pollution, indicating that energy consumption contributes to environmental degradation. Specifically, the positive coefficient, 0.0897, implies that an increase in energy use of 1% within the dataset may lead to increases in NO_2_ pollution of 0.0897%. During the past two decades, rapid economic growth in China has consumed a large amount of energy resources, particularly fossil fuels, with coal playing a dominant role in the energy structure. The overuse of fossil fuels generated more NO_x_ in the air, thus affecting the local environment. The positive coefficient for the total nighttime light data suggests that the government should increase the levels of energy-saving technologies and renewable energy in the energy use structure.

In terms of the urban road area variable, in contrast to the estimated coefficient of the PLS model, it was significant and positive in the fixed effects model, in line with our expectations, implying that the bias had been corrected because the fixed effect has been controlled for in the model. The positive effect of the urban road area (0.0267) on NO_2_ pollution indicates that within the dataset an increase in urban road area of 1% caused a rise of NO_2_ level of 0.067%.

Vegetation coverage has a negative impact on NO_2_ pollution. The negative coefficient, −0.2064, indicates that an increase in the vegetation coverage of 1% within the dataset is associated with a reduction in NO_2_ pollution of 0.2064%. The changes in vegetation coverage largely affect local air quality, thus changing the NO_2_ concentrations. Therefore, increasing the vegetation coverage can reduce NO_2_ concentrations in local city regions.

Meteorological parameters also influence the NO_2_ pollution level. We found that cities with a higher temperature, higher relative humidity, higher wind speed, and lower ambient pressure can reduce the NO_2_ value.

## 4. Conclusions and Policy Implications

The main aim of this research is twofold. First, we assessed the spatial and temporal patterns of tropospheric NO_2_ columns based on OMI satellite instrument observations. In the second stage, we evaluated the driving factors that influence NO_2_ pollution using an extended STIRPAT model. The findings were as follows. The highly NO_2_ polluted regions, which are concentrated in certain city-clusters and polluted regions, expanded significantly during 2005–2012. Tropospheric NO_2_ exhibited highly clustered spatial patterns over China. We found that those factors that had a positive relationship with tropospheric NO_2_ were population, per capita GDP, the share of the secondary industry, ambient air pressure, total nighttime light data, and urban road area, indicating that these influencing factors drive up the NO_2_ pollution level. On the other hand, factors that may lower the NO_2_ pollution level were NDVI, relative humidity, temperature, and wind speed, which all contributed to reducing the environmental pressure caused by an increase in the NO_2_ pollution level. Although the meteorological factors may have a significant influence on the NO_2_ pollution level, they cannot be changed directly. Therefore, a transformation of the patterns of human activity is necessary to mitigate NO_2_ pollution at the city level in China.

Population and affluence are the major influencing factors driving up the NO_2_ pollution level and causing consequent environmental pressures of many kinds. As China’s economy grows and income levels increase, the improvement of living standards has led to increasing demand for energy and resources. The provision of a large number of energy-intensive products and services has also posed a challenge for the environment. Given the fact that China has a large population and is witnessing a rise in income levels, environmental awareness needs to be enhanced. Besides, it is well known that secondary industry is the main contributor to a variety of pollution emissions, and is a major contributing factor to the NO_2_ level. We find that it is necessary to focus on industrial upgrades and a rapid transition from highly-polluting secondary industry to tertiary industry, which is characterized by high added value and less pollution. In addition, regarding the highly polluting and largest NO_2_-emitting sectors, namely, power plants, steel, and cement, de-nitrification systems that involve selective catalytic or non-catalytic reduction are urgently needed in order to further improve the de-nitrification efficiency and thus reduce the environmental pressure caused by NO_2_ emissions from existing sources. With regards to another large source of NO_2_ emissions, residential energy use, environmental education should be improved together with the increase in income levels. In order to sustain the trade-off between economic levels and environmental quality, environmental awareness enhancement and low-emission lifestyles should be encouraged.

## Figures and Tables

**Table 1:** Descriptive statistics for variables.

Variable	Definitions	Unit	Mean	Std. Dev.	Min	Max
*NO_2_*	Tropospheric NO_2_ VCDs	10^15^ molecules cm^−2^	6.76	5.54	0.80	27.86
*Pop*	Population per km^2^	Capita/sq.m	438.12	323.27	15.89	2590.95
*PCGDP*	Per capita gross domestic product	Yuan/Capita	25611.11	20089.61	1652.48	151645.00
*STRatio*	Ratio of secondary industry to tertiary industry	%	1.58	0.90	0.34	9.05
*Road*	Urban road area	10,000 km^2^	1376.74	1752.99	14.84	13322
*NTL*	Nighttime light values	DN	9338853	7473880	499456	48631951
*NDVI*	Normalized difference vegetation index	Unitless	0.57	0.13	0.08	0.78
*Pres*	Ambient air pressure near ground	hPa	969.96	54.60	751.03	1016.86
*Humi*	Relative humidity	%	68	8	42	84
*Temp*	Temperature	︒C	14.31	5.00	0.43	23.72
*WS*	Wind speed	m/s	2.12	0.52	1.09	4.80

**Table 2:** Pearson correlation coefficients.

Variable	*LnNO_2_*	*LnPop*	*LnPCGDP*	*LnSTRatio*	*LnRoad*	*LnNTL*	*LnNDVI*	*LnPres*	*LnHumi*	*LnTemp*	*LnSpeed*
*LnNO_2_*	1										
*LnPop*	0.6828 [0.0000]	1									
*LnPCGDP*	0.4523 [0.0000]	0.1900 [0.0000]	1								
*LnSTRatio*	0.2308 [0.0000]	0.0432 [0.0567]	0.3087 [0.0000]	1							
*LnRoad*	0.5474 [0.0000]	0.5055 [0.0000]	0.5900 [0.0000]	−0.0776 [0.0006]	1						
*LnNTL*	0.5271 [0.0000]	0.2748 [0.0000]	0.4453 [0.0000]	−0.0344 [0.1290]	0.6473 [0.0000]	1					
*LnNDVI*	−0.0231 [0.3079]	0.3119 [0.0000]	−0.2944 [0.0000]	−0.2389 [0.0000]	−0.0732 [0.0012]	−0.0702 [0.0020]	1				
*LnPres*	−0.0417 [0.0000]	0.4341 [0.0000]	-0.0841 [0.0002]	−0.1443 [0.0000]	0.0449 [0.0476]	−0.1092 [0.0000]	0.7222 [0.0000]	1			
*LnHumi*	−0.1070 [0.0000]	0.4213 [0.0000]	−0.1720 [0.0000]	−0.1487 [0.0000]	−0.0002 [0.9942]	−0.2034 [0.0000]	0.7325 [0.0000]	0.8837 [0.0000]	1		
*LnTemp*	0.1649 [0.0000]	0.5543 [0.0000]	−0.0529 [0.0197]	0.0818 [0.0003]	0.0558 [0.0138]	−0.1430 [0.0000]	0.3881 [0.0000]	0.6836 [0.0000]	0.5866 [0.0000]	1	
*LnWS*	0.2446 [0.0000]	0.0355 [0.1178]	0.3530 [0.0000]	0.0063 [0.7804]	0.2890 [0.0000]	0.4073 [0.0000]	−0.3701 [0.0000]	−0.2689 [0.0000]	−0.3503 [0.0000]	−0.3162 [0.0000]	1

Note: *p*-values in brackets.

**Table 3:** Pooled least squares results.

Variable	Coefficient	Std. Err	Probability	VIF	Tolerance
*LnPop*	0.7449	0.0176	0.0000	2.54	0.3946
*LnPCGDP*	0.2400	0.0195	0.0000	2.23	0.4476
*LnSTRatio*	0.1976	0.0248	0.0000	1.43	0.7014
*LnRoad*	−0.0442	0.0167	0.0080	3.19	0.3130
*LnNTL*	0.2004	0.0174	0.0000	2.18	0.4589
*LnNDVI*	0.5498	0.0538	0.0000	3.08	0.3250
*LnPres*	−0.2134	0.0410	0.0000	7.21	0.1386
*LnHumi*	−2.1780	0.1679	0.0000	5.80	0.1725
*LnTemp*	0.0172	0.0332	0.6050	2.97	0.3366
*LnWS*	−0.0273	0.0477	0.5670	1.60	0.6252
*Constant*	−8.4091	0.2605	0.0000		
*R^2^*	0.7514				
*Adj R^2^*	0.7501				
*F-Statistic*	584.35				
*p-value (F-Statistic)*	0.0000				

**Table 4:** Results of the fixed effects and random effects models.

	Fixed Effects Model			Random Effects Model		
Variable	Coefficient	Std. Err	Probability	Coefficient	Std. Err	Probability
*LnPop*	0.3874	0.1032	0.0000	0.6855	0.0346	0.0000
*LnPCGDP*	0.3783	0.0163	0.0000	0.3456	0.0151	0.0000
*LnSTRatio*	0.1055	0.0377	0.0050	0.1386	0.0325	0.0000
*LnRoad*	0.0267	0.0135	0.0480	0.0158	0.0131	0.2280
*LnNTL*	0.0897	0.0233	0.0000	0.1245	0.0202	0.0000
*LnNDVI*	−0.2064	0.0818	0.0120	−0.1316	0.0648	0.0420
*LnPress*	0.0439	0.0239	0.0660	0.0240	0.0231	0.2980
*LnHumi*	−0.6783	0.1272	0.0000	−0.9016	0.1222	0.0000
*LnTemp*	−0.1592	0.0499	0.0010	−0.2156	0.0399	0.0000
*LnSpeed*	−0.4169	0.0667	0.0000	−0.3399	0.0581	0.0000
*Constant*	−5.7434	0.6871	0.0000	−7.5706	0.3597	0.0000
*R^2^*	0.5712			0.5655		
*F-Statistic/Wald Statistic*	225.24			2805.35		
*p-value*	0.0000			0.0000		
